# Young‐onset dementia diagnosis, management and care: a narrative review

**DOI:** 10.5694/mja2.51849

**Published:** 2023-02-19

**Authors:** Samantha M Loi, Monica Cations, Dennis Velakoulis

**Affiliations:** ^1^ University of Melbourne Melbourne VIC; ^2^ Royal Melbourne Hospital Melbourne VIC; ^3^ Flinders University Adelaide SA

**Keywords:** Dementia, Biomarkers, Diagnostic imaging, Neuropsychiatry

## Abstract

- Young‐onset dementia comprises a heterogeneous range of dementias, with onset at less than 65 years of age. These include primary dementias such as Alzheimer disease, frontotemporal and vascular dementias; genetic/familial dementias; metabolic disorders; and secondary dementias such as those that result from alcohol use disorder, traumatic brain injury, and infections.
- The presentation of young‐onset dementia is varied and may include cognitive, psychiatric and neurological symptoms. Diagnostic delay is common, with a frequent diagnostic conundrum being, “Is this young‐onset dementia or is this psychiatric?”.
- For assessment and accurate diagnosis, a thorough screen is recommended, such as collateral history and investigations such as neuroimaging, lumbar puncture, neuropsychology, and genetic testing.
- The management of young‐onset dementia needs to be age‐appropriate and multidisciplinary, with timely access to services and consideration of the family (including children).

Young‐onset dementia (YOD) refers to any dementia where symptom onset occurs at less than 65 years of age^1^ and is distinct from childhood dementia, which is often diagnosed at 14 years of age or less.^2^ YOD accounts for about 5% of all dementias, with an estimated prevalence of 119 per 100 000.^3^ In Australia, about 28 000 people are living with YOD,^4^ with major associated personal, psychosocial and economic costs.^5^ While the concept of “young‐onset dementia” has been used since the 1990s, it is only in the past decade that there has been increasing recognition that the diagnostic and post‐diagnostic issues in YOD are distinct from those occurring in older people. People with YOD contend with delays to diagnosis and difficulty navigating the complex health, aged and disability care systems, while often also caring for or supporting children or young people. Improved access to assessment, timely diagnosis, and appropriate post‐diagnosis care can have important benefits for improving function and quality of life. In this narrative review, we present a clinical approach to YOD, reporting on advances in diagnosis, management, and access to care. Using our collective knowledge of YOD, combined with a PubMed search of “young‐onset dementia” and “younger‐onset dementia”, we included publications from 2002 to 2022, prioritising those from an Australian setting. We included what we considered seminal papers in the area and excluded childhood dementia and secondary causes of dementia.

## Aetiology and diagnosis

As for dementias with an older onset, Alzheimer disease (AD), frontotemporal dementias (FTD)^6^, ^7^ and vascular dementias^8^, ^9^ are the most frequent causes of YOD. However, the aetiological profile of YOD is more diverse than in later life and is more likely to be caused by a rarer pathology or occur secondarily to another condition. In particular, alcohol‐related dementias^10^ usually have an onset in midlife. YOD can also occur secondarily to other causes, including neurological (eg, Huntington disease, Parkinson disease, multiple sclerosis, traumatic brain injury, motor neuron disease), infective (eg, neurosyphilis, human immunodeficiency virus) and other (eg, systemic lupus erythematosus, alcohol‐related).^1^ People with intellectual disability are recognised as having increased risk for developing dementia,^11^ and most people with Down syndrome will exhibit symptoms consistent with AD by 65 years of age.^12^ For Indigenous Australians, the prevalence of dementia is reported as 12.4%. This is five times greater than the Australian population rate (2.4%), with a much earlier onset of illness.^13^ The most common cause of dementia in an Indigenous Australian population was reported as dementia not otherwise specified (53%) and Alzheimer type (24%).^13^

The presenting symptoms of YOD are more variable than the initial symptoms seen in older adults. In younger people, early presenting symptoms often include behaviour, language and personality change, and executive dysfunction (Box 1). In young‐onset AD, there are several variants: (i) posterior cortical atrophy presenting with visual problems (ocular motor apraxia, simultanagnosia), apraxias and Gertsmann syndrome (acalculia, finger agnosia, agraphia, left–right disorientation); (ii) frontal variant AD or behavioural/executive AD, presenting with symptoms similar to behavioural variant FTD (bv‐FTD);^14^ and (iii) logopaenic variant primary progressive aphasia, presenting with language difficulties such as word‐finding difficulties and decreased repetition of sentences.^15^ FTD often presents with impulsive or inappropriate behaviour, executive problems, and language impairments.^16^ The most common genetic cause of FTD, the GGGGCC hexanucleotide repeat mutation in *C9orf72*,^17^ can present with symptoms consistent with a bv‐FTD or motor neuron disease (or both), as well as psychiatric symptoms that may predate cognitive symptoms by up to two decades.^18^

Box 1 **The aetiology of young‐onset dementia**

**Table:** 

Dementia type	Initial symptoms	Genes implicated	Proteins implicated	Reference
Alzheimer disease	Short term memory impairment Word‐finding difficulties Disorientation	*APOE* *PSEN1* *PSEN2* *APP*	Amyloid‐β Tau	McKhann et al^19^
Posterior cortical atrophy	Apraxias Visual perception impairment Alexia Gerstmann syndrome		Amyloid‐β Tau	
Behavioural variant FTD	Behavioural and personality changes Loss of empathy Disinhibition Changes in appetite Apathy Repetitive or stereotyped behaviours Rigidity	*MAPT* *GRN*	Tau TAR DNA‐binding protein 43	Rascovsky criteria^20^
FTD–MND	Behavioural changes Motor changes: weakness, upper and lower motor neuron signs	*C9orf72*	TAR DNA‐binding protein 43 RNA‐binding protein FUS	
Semantic dementia	Semantic impairment Anomia Single word comprehension impairment		Tau	Gorno‐Tempini et al^15^
Progressive non‐fluent aphasia	Progressive speech production impairment Halting, slow, effortful speech Motor speech apraxia		Tau	Gorno‐Tempini et al^15^
Progressive supranuclear palsy	Falls, gait and balance problems Vertical supranuclear gaze palsy		Tau (four repeats)	
Corticobasal syndrome	Asymmetrical apraxia and rigidity Alien limb phenomenon		Tau (four repeats)	Armstrong et al^21^

FTD = frontotemporal dementia; MND = motor neuron disease.

This variability highlights the difficulty with timely and accurate diagnosis resulting in diagnostic delay of three to five years.^22^, ^23^ This is longer than time to diagnosis in older‐onset dementia.^24^ An accurate diagnosis of dementia early in the disease process is critically important for younger people as there are significant implications for management, access to support services, prognosis, and future planning. Guidelines recommend consideration of the possibility of YOD in a middle‐aged individual who presents with reasonably new onset psychiatric symptoms with no previous psychiatric history.^25^ Other reasons for referral for further investigation and assessment are listed in Box 2.

Box 2 **Red flags and factors that may require further assessment for young‐onset dementia**

- New onset (ie, in past 10 years rather than development in adolescence or young adulthood) in someone of middle age who has no previous psychiatric history
- Behaviour changes that are not consistent with previous personality
- Progressive neurological symptoms or seizures
- Cognitive/behavioural/psychiatric changes in a person with a family history of young‐onset dementia
- Minimal improvement of psychiatric symptoms following psychotherapy, counselling or psychotropic medications

International consensus on guidelines for assessment in YOD is listed in Box 3.^25^ Due to the potential reversibility of secondary YODs, it is important to perform a thorough screen for reversible causes (Box 4). The Mini‐Mental State Examination (MMSE)^26^ may be within normal limits for an individual with a YOD such as bv‐FTD,^27^ and an alternative screening cognitive examination (such as Addenbrooke's Cognitive Examination III Revised^28^ or Neuropsychiatry Unit Cognitive Screening [NUCOG] tool^29^) should be considered. Identification of dementia in people with Down syndrome and other intellectual disabilities can be difficult without premorbid cognitive assessment data. Neuropsychological assessment of a person with intellectual disability in early adulthood can be useful for detecting cognitive decline,^30^ and mid‐ to late‐life behaviour changes reported by family and carers can be important early signs of neurodegeneration.^31^ For Indigenous Australians, the Kimberley Indigenous Cognitive Assessment is a reliable cognitive assessment tool for detecting cognitive impairment and can be used in rural and remote areas such as in the Kimberley, Northern Queensland and the Pilbara.^32^

Box 3 Guidelines for assessment in young‐onset dementia*

**Table:** 

Aspect of care	Recommendations
Pre‐assessment and communication	Many professionals required over time
	Important to convey the diagnosis to the individual and family and remain open to review and modify this diagnosis
	Important to obtain rapport
	Need to find out what supports are required
	Checking if the individual has capacity
History taking	Collateral history important
	Symptom onset and type
	Frontotemporal dementia symptoms (such as loss of empathy, apathy, behavioural changes)
	Physical health and other medical conditions
	Function: eg, activities of daily living
	Drug and alcohol history
Family history	Obtain a three‐generational history of young‐onset dementia
Physical examination	Including neurological examination
Risk assessment	Occupational risks, driving, other risky behaviour eg, gambling
Psychiatric assessment	Previous psychiatric history and symptoms
	History of learning disability or intellectual disability
Neuroimaging	Magnetic resonance imaging (at the minimum)
Neuropsychological assessment	Screening testing, not just Mini Mental State Examination (eg, ACE‐R or NUCOG)
Palliative care	Support is required from diagnosis to end of life care

ACE‐R = Addenbrooke's Cognitive Examination Revised; NUCOG = Neuropsychiatry Unit Cognitive Screening tool.

* Adapted from O'Malley et al.^25^

Box 4 **Potential reversible causes of dementia occurring in a younger person**

- Inflammatory: multiple sclerosis, neurosarcoidosis, autoimmune encephalitis
- Infection: human immunodeficiency virus, neurosyphillis, Whipple disease
- Misuse of alcohol or other drugs
- Heavy metal poisoning
- Metabolic and endocrine: thyroid disease, Wilson disease
- Other causes: obstructive sleep apnoea, normal pressure hydrocephalus

### Imaging

Brain neuroimaging, including structural and functional scans, forms part of a comprehensive assessment for YOD.^25^ A magnetic resonance imaging (MRI) brain scan may detect atrophy in regional areas indicative of a dementia syndrome. For example, hippocampal atrophy is seen in AD and frontotemporal atrophy in FTD. Radiological visual rating scales can be a quick, relatively accessible and practical way of improving diagnostic accuracy, although this may depend on clinical experience,^33^ and automated normative morphometry, if available, may improve detection of regional atrophy regardless of radiological experience.^34^ A neurodegenerative protocol for brain MRI would ideally include the three main planes (sagittal, coronal and axial) and include T1, T2, fluid‐attenuated inversion recovery and diffusion weighted imaging sequences in each. Functional imaging such as fluorodeoxyglucose positron emission tomography (FDG‐PET) or single‐photon emission computed tomography scans, less accurate than FDG‐PET, can identify dementia‐related changes by quantitatively demonstrating cerebral glucose hypometabolism and decreased brain activity, respectively.^35^ Box 5 summarises common neuroimaging changes in various dementias.

Box 5 **Neuroimaging changes in some young‐onset dementias**

**Table:** 

Young‐onset dementia	Magnetic resonance imaging: areas affected	Positron emission tomography: areas affected	Other imaging modalities
Alzheimer disease	Hippocampus Medial temporal lobes	Precuneus Posterior cingulate Parietotemporal	Amyloid‐β positron emission tomography
Posterior cortical atrophy	Posterior/occipital regions	Parietooccipital cortices	Amyloid‐β positron emission tomography
Behavioural variant frontotemporal dementia	Frontal (medial, orbitofrontal, anterior insula)	Frontotemporal	
Semantic frontotemporal dementia	Anterior temporal	Frontal midline structure (cingulate, orbitofrontal and anterior medial cortices, insula)	
Progressive non‐fluent aphasia		Frontotemporal region of left side: inferior frontal region, dorsolateral prefrontal, Broca and Wernicke area, and inferior temporal regions

New molecular neuroimaging research technologies can provide a non‐invasive method of improved diagnostic accuracy. Amyloid‐β (Aβ) imaging can selectively identify the Aβ‐containing plaques that are the hallmark pathological feature of AD.^36^ Several compounds are available to accurately distinguish AD from age‐matched controls and can assist in the distinction between different types of dementia such as AD and FTD.^37^ The degree of amyloid accumulation on Aβ imaging may not correlate with severity of cognitive impairment, as the accumulation of amyloid likely reaches a threshold.^37^ A negative Aβ PET scan in a person with cognitive symptoms is helpful for differential diagnosis, because it excludes the presence of amyloid as a cause of these symptoms and can exclude AD. Tau PET scans have become useful in identifying the tau based neurofibrillary tangles, which are pathological contributors to AD.^36^ These PET ligands are not specific for AD as they also identify tau accumulation in other tauopathies such as some FTDs, progressive supranuclear palsy, and chronic traumatic encephalopathy.^38^ Regional distributions of tau can help to guide differential diagnosis.^39^

### To lumbar puncture or not to lumbar puncture?

Analysis of cerebrospinal fluid (CSF) via lumbar puncture is another important tool in the diagnosis of dementia in younger people.^25^ The CSF can be analysed to determine the quantity of Aβ40 and Aβ42 and total tau (t‐tau) and phosphorylated tau (p‐tau), both indicative of AD. Although a CSF analysis via lumbar puncture may be more invasive than a brain scan, it is potentially more cost‐effective and accessible.^40^ CSF analysis can rule in AD and identify other neurological disorders that might also cause symptoms of confusion (eg, tumours and infections). There is very strong agreement between the CSF ratios of Aβ40 and Aβ42 with t‐tau and p‐tau with amyloid PET, with negative predictive values of at least 98% and positive predictive values of at least 77%.^41^ This suggests that CSF analysis for AD proteins has similar correlation in the quantification of amyloid pathology, further supporting the use of lumbar punctures, especially in younger people. The underuse of CSF analysis^40^ may be due to physician hesitancy in ordering a lumbar puncture, lack of confidence in performing one, perceived risk of side effects, and a perception that there are limited benefits of diagnosing AD given a lack of disease‐modifying treatments.^40^ In addition, CSF analysis for Alzheimer proteins requires specialised laboratories and standardised procedures, which are not easily accessible all over Australia and currently are not funded by Medicare. Despite this, given the safety, tolerability, patient acceptability and diagnosis that a lumbar puncture can provide, CSF analysis should be part of the assessment process for those suspected of having YOD.^25^, ^40^

Recent research evidence suggests that neurofilament light chain, a component of the axons that is released into CSF when there is damage (eg, due to neurodegeneration or traumatic brain injury), may have utility in the diagnosis of YOD. Analysis of neurofilament light chain has been found to have 87% sensitivity and 90% specificity in differentiating between primary psychiatric disorders and dementia in younger people,^42^ and this can also be replicated with plasma biomarkers of neurofilament light chain, p‐tau 181, p‐tau 217, glial fibrillary acidic protein, Aβ40 and Aβ42,^43^ potentially improving accessibility.

### Genetic factors

About 15% of all YOD cases are caused by an autosomal dominant genetic mutation.^44^ Genetic testing is highly recommended when dementia is diagnosed in a younger person, especially with a family history of YOD,^25^ onset before 45 years of age, and with a dementia type with a high heritability rate. Directly inherited cases occur in nearly 50% of people with onset before age 45 years.^45^ FTD is also more likely to be inherited than other dementia types, with autosomal‐dominant inheritance occurring in 20–50% of cases (usually the *C9orf72* mutation).^46^ Other familial types of YOD include Huntington disease, Niemann–Pick disease type C, and cerebral autosomal dominant arteriopathy with subcortical infarcts and leukencephalopathy.

Finding a genetic cause of dementia can provide diagnostic certainty and has significant implications for the family of the affected individual. Three types of next generation sequencing techniques are currently available for clinical use: (i) targeted gene panels that only include specific genes for a particular disorder; (ii) whole exome sequencing that can read sequences from all coding regions; and (iii) whole genome sequencing that analyses sequences in coding and non‐coding regions (this may include all genetic causes of dementias and could potentially increase diagnostic accuracy).^47^, ^48^ There are limitations with this technology in that large duplications and structural variations cannot be read by these techniques, nor can triplicate repeat disorders such as the CAG (cytosine, adenine, guanine) repeat in Huntington disease and disorders related to the *C9orf72* mutation.^48^ In addition, not all genetic variants have clinical significance, and some genetic risk factors, such as the *APOE* risk allele, have unclear significance. Genetic counselling before testing and receiving results is therefore recommended for discussion of this complex information for families.^48^

## Management

People with YOD usually live for many years after their diagnosis and longer than those with older‐onset dementia.^49^ The delivery of high quality, integrated and multidisciplinary post‐diagnosis management and care promotes quality of life and community engagement for the benefit of the person and their wider family and community.

### Medical management

Medical management in the first years after a YOD diagnosis should include repeat assessments, including neuroimaging and neuropsychology, to characterise the dementia (ie, diagnostic stability, prognosis). Some dementias, such as bv‐FTD, are difficult to diagnose at one timepoint and individuals may perform within normal limits on neuropsychological testing, so are often misdiagnosed with a psychiatric condition, a different dementia,^50^ or phenocopy syndrome (people presenting with the neuropsychiatric symptoms that mimic bv‐FTD but who may not have frontotemporal atrophy or hypometabolism on neuroimaging, or the progression/deterioration required for a diagnosis of dementia).^51^ There are recommendations for differentiating bv‐FTD from psychiatric disorders,^27^ and regardless of the type of YOD, repeated assessments are important to confirm or specify a diagnosis.^50^

For young‐onset AD, treatment using cholinesterase inhibitors (donepezil, galantamine, rivastigmine) and memantine are indicated but do not modify disease progression.^52^ Management of cardiovascular risk factors and other lifestyle modification may also slow cognitive decline in people with YOD.^53^

Medical management is important for addressing the psychiatric symptoms and impacts of YOD. Most people with YOD experience symptom onset at a time of peak financial, familial and occupational responsibility, and their illness commonly causes profound distress. People with YOD regularly report loss of identity and role changes, feelings of hopelessness and powerlessness, and social exclusion.^54^, ^55^ Recent research has found that people with YOD aged 45 to 64 years are 2.8 times more likely to die by suicide than people without dementia in the same age range.^56^ Although this likely represents a small number of suicide deaths overall, the risk for death by suicide declines with time from diagnosis. As such, close follow‐up soon after a diagnosis is given is recommended. Treatment for low mood and anxiety should follow general treatment guidelines, including modified psychotherapy and antidepressants with short term benzodiazepines for depression and anxiety.^57^ Sleep disturbances can be managed by non‐pharmacological management tailored to the individual, and melatonin or short term low dose benzodiazepines may be required.^58^

### Post‐diagnosis care and support

In Australia, most dementia care services are embedded within the aged care system, and as such are designed and tailored for older people living with dementia.^59^, ^60^ These services can lack suitability for young people with dementia and their families.^60^ The transferral of funding arrangements for social and allied health care services for YOD to the National Disability Insurance Scheme (NDIS) in 2017 was therefore welcomed by people with YOD and their families. However, recent research suggests that people with YOD and their families continue to have difficulty accessing the NDIS and finding suitable services with which to spend their funding.^61^

Post‐diagnostic care for younger people with dementia should be tailored, flexible, affordable and provide meaningful engagement.^60^ An assessment of the specific disabilities experienced by the person will help to guide non‐pharmacological treatment and care. People with YOD may benefit from a multidisciplinary approach including psychology or counselling, functional assessment with home modifications and help with activities of daily living (known as Supported Independent Living within the NDIS), speech pathology, physiotherapy and/or exercise physiology, home support (eg, cleaning, cooking), financial management, and assistance with advance care planning. Advance care planning should begin at the time of diagnosis and include consideration of financial affairs, care and living arrangements, end of life preferences, and eventual driving cessation. Some people with YOD with a shorter life expectancy (eg, motor neuron disease, some FTDs) may consider voluntary assisted dying if this is available in their state. Consideration should be given to the specific eligibility requirements for voluntary assisted dying that differ between states. Social support, particularly with other younger people with rare illness, can promote quality of life.^62^ Multi‐agency support is often needed to promote the continued employment of younger people with dementia, starting with accommodations and changes in role, before transitioning into early retirement.^63^

### Behaviour management

Neuropsychiatric and behavioural symptoms are important causes of distress for people with YOD^64^ and their family members,^65^ and can cause premature entry to residential care.^66^ These symptoms occur in most individuals with YOD and can be categorised into affective (depression, apathy, anxiety, elation, irritability); psychotic (delusions, hallucinations, paranoia); hyperactivity (pacing, restlessness, disinhibition); aggression/agitation (verbal, physical); and other changes (wandering, intrusiveness, shadowing, reverse sleep–wake cycle, inappropriate behaviours such as urination).^66^ Complex neuropsychiatric and behavioural symptoms can lead a person with YOD to “bounce” between aged, disability and health services because of a lack of multidisciplinary management skills and services.^67^ Challenging behaviour and personality change is particularly common among those with frontal‐related dementias such as bv‐FTD, alcohol‐related dementias, and chronic traumatic encephalopathy.^68^

In some cases, behaviour changes are manifestations of an unmet need that the individual is unable to communicate (eg, temperature regulation, boredom) or a reflection of the person's lowered stress threshold.^69^ Recommendations for the management of behavioural symptoms endorse non‐pharmacological strategies as a first line approach.^70^ Assessment of the triggers, features and consequences of a behaviour can help to identify its function for the person and point to potentially effective interventions.^69^ Psychoeducation and training for family carers about behaviour management can effectively reduce the impact of behaviour change.^69^

Where pharmacological treatment is required as an adjunct to non‐pharmacological therapies, international guidelines recommend a “start low and go slow” approach to prescribing.^71^ Benzodiazepines may provide short term benefit for sleep disturbance, agitation and anxiety.^57^ Pain is a common contributor to behaviour change, and pain management should be considered as a useful addition.^72^ Risperidone is indicated for short term (maximum 12 weeks) management of severe psychotic symptoms or aggressive behaviour dementia,^73^, ^74^ but prescribers should consider that all antipsychotic medications are associated with major risks for people with dementia, including stroke and extrapyramidal symptoms.^75^ There is a black box warning for some antipsychotic medications with risk of sudden death due to cardiac arrythmias, although this has been reported for those over 65 years of age.^76^ However, obtaining an electrocardiogram before commencement is recommended for those who have dementia and are aged under 65 years. Policy and regulatory efforts to reduce the overuse of antipsychotic medications are underway in many countries.^77^ Prescribing should also consider that some behaviour changes are less responsive to psychotropic medications, including wandering, vocalisations, shadowing, intrusiveness, repetitive activities, and inappropriate behaviours such as urination/defecation, undressing, and sexualised behaviour.^69^, ^78^

### Family

YOD has a major impact on the family of the person affected. Family carers of people with YOD experience changes to their self‐identity and relationships, and are at high risk for social isolation.^54^, ^79^ Carer burden is high, particularly for spouses of people with YOD, who are sometimes also caring for children and ageing parents.^80^, ^81^, ^82^ Children of people with YOD experience disrupted emotional, psychological and social development, and are at high risk for the development of psychological illness.^83^ A whole‐of‐family approach to the management of YOD is needed to ensure that families have adequate access to financial resources, respite, social and educational opportunities, and psychoeducation.^84^ Carer and family wellbeing can be promoted via psychological or counselling support, regular respite breaks, NDIS pre‐planning and support coordination, and social support groups such as those offered by Dementia Australia.

### Housing

Although younger people with dementia usually prefer to live and die at home, cognitive, functional and behaviour impairments can necessitate long term care. Traditional residential care facilities for older adults may be inappropriate for younger people and the Australian Government has pledged that no one aged under 65 years will enter or live in residential aged care by 2022 and 2025, respectively.^85^ However, few appropriate long term housing alternatives currently exist for people with YOD, and the establishment of YOD‐specific facilities may be financially untenable, particularly in regions where few people with YOD live. Potential solutions might include supported disability accommodation designed to accommodate cognitive disabilities, and/or hybrid models that capitalise on both the extensive experience in dementia care within the aged care sector and the strengths‐based focus of the disability sector.^67^

### Prognosis

Accurate differential diagnosis of YOD is helpful for estimating progression and prognosis, and the course of YOD will depend on the dementia type, premorbid health, and other factors. Young people with AD experience faster cognitive decline than older people with AD, but research examining the impact of onset age on symptom progression in other dementias is inconclusive.^86^

Recent research found that the median survival from age of onset for younger people with AD, FTD and vascular dementia was 11.3 years, 10.6 years and 12.3 years, respectively (Loi SM, Tsoukra V, Sun E, et al. Manuscript in preparation). Dementias caused by autosomal‐dominant gene mutations (eg, *MAPT*‐type FTD) are usually associated with earlier age of onset and age at death,^87^ and FTD associated with motor neuron disease has the shortest duration of survival.^88^ Overall, people with YOD lose 10 to 15 years of life expectancy.^89^

## Conclusion

Our understanding of the pathogenesis, aetiological profile, diagnosis and management of YOD has significantly improved over the past decade. We propose several actions to improve the care of people with YOD in Australia (Box 6). Younger people with dementia may live for a decade with the disease and most will remain living in the community, supported by families and community services. Thus, we also propose that a paradigm shift is needed with the concept of “dementia”, in order to decrease stigma and fear associated with this condition. Dementia ought to be considered a chronic disease which progresses over time, but with age‐appropriate interventions and services, we can continue to support and improve the quality of life of people with dementia as they deteriorate. Although there are no disease‐modifying treatments for most types of YOD, timely diagnosis and multidisciplinary care and support can have major benefits for promoting community participation and quality of life for people with YOD and their families.

Box 6 **Actions to improve the care of people living with young‐onset dementia and their families**

- Promote pathways of care for timely diagnosis, especially for Indigenous Australians, including integration of the disability, health and aged care sections, and access to telehealth
- Improve diagnostic accuracy
- Improve pathways to research so that people with young‐onset dementia can be included
- Highlight the concept that young‐onset dementia is a condition that causes disability that requires timely access to the National Disability Insurance Scheme (NDIS), and to improve the NDIS workforce in knowledge and care of younger people with dementia
- Promote prompt advanced care planning, consideration of driving cessation, and implementation of accommodations to promote ongoing workforce participation
- Integrate age‐appropriate services for family members of people with young‐onset dementia in existing health care systems
- Enable access to appropriate accommodation and palliative care

## Open access

Open access publishing facilitated by The University of Melbourne, as part of the Wiley ‐ The University of Melbourne agreement via the Council of Australian University Librarians.

## Competing interests

No relevant disclosures.

## Provenance

Commissioned; externally peer reviewed.
